# Long-term intake of *Lilium lancifolium* mitigated osteoarthritic effects by suppressing inflammatory cytokines in a dog model

**DOI:** 10.14202/vetworld.2022.2012-2020

**Published:** 2022-08-22

**Authors:** Jeong-Hwi Cho, Yang-Gyu Park, Jinyoung Choi, Gareeballah Osman Adam, Eun-Myeong Ju, Ho Park, Hong-Geun Oh

**Affiliations:** 1 R&D Division, HUVET Co. Ltd., Iksan-si 54531, Republic of Korea; 2 Department of Veterinary Medicine and Surgery College of Veterinary Medicine, Sudan University of Science and Technology, Hilat Kuku, Khartoum 11311, Sudan; 3 Department of Clinical Laboratory Science, Wonkwang Health Science University, Iksan 54538, Republic of Korea

**Keywords:** articular cartilage, inflammation, joint pain, *Lilium lancifolium*, MMP-9, osteoarthritis

## Abstract

**Background and Aim:**

Osteoarthritis (OA) is a chronic, painful, degenerative inflammatory disease of the synovial joints. Regular use of nonsteroidal anti-inflammatory drugs to decrease OA pain can have severe side effects, such as gastric irritation, ulcers, and heart problems. Natural products are extensively used to minimize OA-associated pain and inflammatory reactions. *Lilium lancifolium* is commonly used to alleviate several diseases through its anti-inflammatory effects. This study examined the impact of *L. lancifolium* extract on alleviating pain and inflammation associated with articular cartilage damage.

**Materials and Methods:**

Hydro-ethanol extracts of the *L. lancifolium* bulb were used. The experimental animals (adult beagle dogs) were divided into four groups: sham, which received neither treatment nor surgery; placebo, which received an empty gelatin capsule; glucosamine, which received glutamine (60 mg/kg); and *L. lancifolium*, which received an *L. lancifolium* extract-filled (60 mg/kg) gelatin capsule for 8 weeks. OA was induced by an expert orthopedic surgeon in 2-year-old dogs through resection of cranial cruciate ligament and lateral collateral ligament. Inflammatory cytokines, enzymes, lameness score, radiology, and histological changes were assessed.

**Results:**

Our experiments showed that long-term oral therapy with *L. lancifolium* alleviated inflammation and increased histological damage. *L. lancifolium* treatment effectively reduced cytokines, such as interleukin-6, metalloproteinase-9, leukotriene-4, prostaglandin, and cyclo-oxygenase in dogs with OA, suggesting the potential to minimize inflammatory reactions in OA. *L. lancifolium* showed anti-inflammatory qualities in dogs with OA. This effect was comparable with that of glucosamine OA treatment.

**Conclusion:**

*L. lancifolium* supplementation represents a possible therapeutic and management option in this model of OA.

## Introduction

Osteoarthritis (OA) is a degenerative joint disease that causes pain, joint stiffness, and muscle atrophy. It is often accompanied by cartilage erosion, synovial inflammation, subchondral osteosclerosis, and osteophyte formation [1–4]. OA is the most common age-related form of arthritis. It is presently defined as an incurable disease. In addition, OA prevalence increases with advanced age, especially in the older population [5–7]. OA prevalence is high in dogs [8, 9], with 20% of dogs aged over 1 year showing varying degrees of OA [10, 11]. OA is often associated with chronic pain, claudication, dysfunction, and poor quality of life, eventually causing loss of joint function and mobility [12].

The overarching goal of OA treatment is to alleviate pain. Pain management restores joint strength and function and maintains the mobility of the affected limb [13, 14]. Nonsteroidal anti-inflammatory drugs (NSAIDs) are usually prescribed to decrease the pain and inflammation associated with OA [12]. NSAIDs act by inhibiting cyclo-oxygenase (COX) and reduce the concentration of pro-inflammatory prostaglandins (PGEs). However, long-term NSAID use can have side effects such as gastric irritation, ulcers, platelet damage, and cardiovascular disease risk [15, 16]. Although NSAIDs such as flunixin meglumine have been indicated to reduce oxidative stress in the laboratory, they have also been shown to delay wound healing [17, 18]. Therefore, studies assessing the role of different substances that can substitute or supplement the limitations of NSAIDs used in OA treatment are in progress. The role of matrix metalloproteinase (MMP)-9 in the pathogenicity of OA is well known [19]. MMP-9 is a significant component of the MMP family involved in collagen and gelatin degradation [20].

*Lilium lancifolium*, a medicinal herb, is a perennial plant that grows in mountains and fields. It is native to China, Japan, Korea, and the Russian Far East. This plant is approximately 1.5 m long, with round scales measuring 5–8 cm in diameter [21]. *L. lancifolium* exhibits anti-inflammatory effects by lowering inflammatory cytokines [22, 23] and scavenging free radicals [24]. The previous studies using interleukin (IL)-1b-stimulated human chondrosarcoma cells have indicated that *L. lancifolium* treatment can effectively inhibit the release of pro-inflammatory cytokines. Rat models of OA induced by monosodium iodoacetate showed *L. lancifolium*’s therapeutic role in OA in minimizing pain and inflammatory responses [25]. Accumulating evidence suggests that *L. lancifolium* regulates inflammation. However, the potential therapeutic role of *L. lancifolium* in canine OA remains unclear.

Therefore, this study investigated the efficacy of *L. lancifolium* in a canine model of OA using radiography, histology, and inflammatory cytokines.

## Materials and Methods

### Ethical approval

All experimental protocols were approved by the Institutional Animal Care and Use Committee of Jeonbuk National University based on ethical procedures and scientific care (approval no. JBNU 2021-035).

### Study period and location

The study was conducted from February 2021 to October 2021 at the HUVET Animal Facility in Iksan City, South Korea.

### Preparation of *L. lancifolium* extracts

Dried *L. lancifolium* bulbs were obtained from Human Herb Co., Ltd. (Deagu, Korea) and pulverized to a suitable size. Then, 50% ethanol (5–10 times the weight of the bulbs) was added to the extraction vessel and stirred at 50°C. Before being lyophilized into powder, the acquired material was concentrated in a rotary evaporator. A hard gelatin capsule was filled with the lyophilized powder (450 mg). Hy Co. Ltd. provided the placebo and *L. lancifolium* used in the experiment (Seoul, Korea).

### Experimental animals and treatment

Two-year-old male beagle dogs with a mean body weight of 10.9 kg (range, 9.4–12.8 kg) were purchased from a local vendor (Orientbio Co. Ltd., Seongnam, South Korea). The dogs were specially bred for this research. Before beginning this study, the dogs were acclimated to the housing facilities for 7 days. They were housed in a standard state with adequate temperature (24 ± 2°C), humidity (60% ± 10%) control, and a 12 h light/12 h dark cycle. They were provided with feed and water twice daily, in the morning and evening.

**Figure-1:**
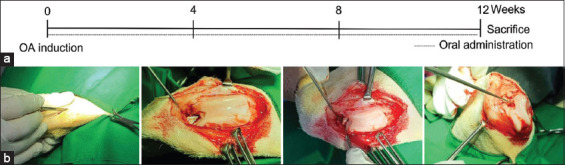
Experimental scheme of OA induction in dogs. Schedule for osteoarthritis induction, and oral *Lilium lancifolium* therapy (a), Cranial cruciate ligament and lateral collateral ligament resection procedure (b). OA=Osteoarthritis.

The experimental animals were divided into four groups (n = 4 per group): Sham, which received neither treatment nor surgery; placebo, which received an empty gelatin capsule and was subjected to surgery; glucosamine, which received glutamine (60 mg/kg) and subjected to surgery; and *L. lancifolium*, which received an *L. lancifolium* extract-filled (60 mg/kg) gelatin capsule after induction of OA. Glucosamine hydrochloride (Sigma-Aldrich, United States) was used as a positive control to compare *L. lancifolium*’s effects. Placebo capsules were used as a vehicle. All test substances were orally administered once daily for 12 weeks (Figure-1).

### Pre-operative assessments

All limbs were evaluated to ensure that there were no pre-existing orthopedic disorders. Before being included in this study, each dog underwent an orthopedic examination by a trained veterinary orthopedic surgeon (week zero). Clinical lameness scores and comfortable range of motion for each dog were determined using modified criteria previously described based on an orthopedic assessment by the same trained orthopedic surgeon [26, 27]. Clinical claudication scores were measured every 2 weeks from week 4 after OA induction, and the sum of clinical lameness scores was recorded for statistical analysis.

### Ligament resection

OA induction and management were performed according to published procedures [28, 29]. The dogs were anesthetized using a ketamine-xylazine mixture (1:1) at 0.1 mL/kg before undergoing aseptic surgery on the right stifle. After draping, each dog was placed in a lateral recumbency position, and a curved parapatellar skin incision was made in the right stifle joint. After separating the fascia from the joint capsule, a cut incision was made in the joint capsule. Along the late fascia incision, the proximal and distal incisions were continued. Laterally, the patella was retracted, and the joint was opened. The cranial cruciate ligament (CrCL) and lateral collateral ligament (LCL) were found and resected using a #11 blade, whereas the joint was fully flexed (Figure-1b). The stifle joint capsule and fascia were closed in a single layer with 2-0 polydioxanone (PDS-II^®^, Johnson and Johnson International, United States). 2-0 Polyglactin 910 was used to close the subcutaneous tissue and skin (Vicryl, ETHICON, United States).

Tramadol (4 mg/kg, IV) and meloxicam (0.2 mg/kg, IV) were administered to the animals 3 days after CrCl/LCL resection. All dogs were returned to their kennels and allowed unrestricted activity in the residential facility. Furthermore, all dogs were required to walk on a leash for 10 min daily to enhance their recovery.

### Orthopedic examination

Each dog underwent an orthopedic assessment to assess knee clinical lameness and function at weeks 4, 6, 8, 10, and 12 after OA induction. These analyses were conducted at all-time points by a single orthopedic surgeon who was unaware of the treatment.

### Radiographic assessment

A radiographic assessment of the stifle joints was conducted for each dog 3 months after OA induction. All radiographic images were evaluated by one trained clinician blind to treatment, according to the previously described criteria [30].

### Blood analysis and measurement of cytokine levels

Whole blood was obtained from the external jugular vein using a syringe. The blood was centrifuged at 4°C for 15 min at 1000 g to separate the serum. The serum was obtained and stored at –80°C until further analysis. The concentrations of IL-6, COX-2, PGE-2, leukotriene (LTB)-4, and MMP-9 were determined using enzyme-linked immunosorbent assay (ELISA) kits (MyBioSource, San Diego, California, USA) according to the manufacturer’s instructions. Glucose (GLU), blood urea nitrogen (BUN), creatinine (CREA), total protein (TP), albumin (ALB), globulin (GLOB), alanine aminotransferase (ALT), and alkaline phosphatase (ALP) were evaluated using a Hitachi 7020 instrument (Hitachi, Tokyo, Japan).

### Histopathological examination

After blood collection, the animal’s knee was cut, and the joint tissue was decalcified in a 10% formalin solution containing 10% ethylenediaminetetraacetic acid. Following that, the specimen was embedded in paraffin and sectioned to a thickness of 7 mm. Slides were stained with hematoxylin and eosin (H&E), Masson’s trichrome (MT), and Safranin-O (S-O) fast green to observe the destruction of cartilage tissue and the degree of inflammation.

Cartilage damage was analyzed based on the depth and severity of the injury. The Osteoarthritis Research Society International (OARSI) grading system was used to score cartilage changes [31]. The degree of inflammation was scored on a grade of 0–6 based on the degree of histopathology in the tissue: 0, surface intact, cartilage intact; 1, uneven but intact surface; 2, surface discontinuity; 3, vertical fissures; 4, erosion; 5, denudation; and 6, deformation.

### Statistical analysis

Statistical results are shown as mean ± standard error of the mean (SEM) with 95% confidence limits. Data were compared statistically using a one-way analysis of variance with a posttest Bonferroni’s multiple comparison test. All statistical analyses were conducted using the GraphPad Prism 8 (San Diego, California, USA) software package; p < 0.05 was considered statistically significant.

## Results

### Blood biochemistry

To verify the safety of *L. lancifolium* extract in dogs, the serum levels of GLU, BUN, CREA, TP, ALB, GLOB, ALT, and ALP were measured at weeks 0, 4, 8, and 12 of the experiment. As listed in Table-1, the levels of the analytes were kept within the normal range [32] without significant changes among groups and at various time points. This result shows that *L. lancifolium* extract has no hepatic or renal toxic effects.

**Table-1:** Biochemical analysis.

Group	Weeks	GLU (mM/L)	BUN (IU/L)	CREA (mg/dL)	TP (g/dL)	ALB (g/dL)	GLOB (g/dL)	ALT (IU/L)	ALP (IU/L)
Sham	0	101.0 ± 9.2	16.0 ± 1.9	0.70 ± 0.11	7.20 ± 0.81	4.1 ± 0.6	3.1 ± 0.5	66.0 ± 14.9	40.0 ± 7.6
	4	99.0 ± 14.4	20.0 ± 3.1	0.50 ± 0.16	7.30 ± 0.85	3.7 ± 0.4	3.6 ± 0.4	48.0 ± 14.1	40.0 ± 13.6
	8	112.0 ± 10.1	19.0 ± 4.0	0.70 ± 0.21	7.90 ± 0.89	4.1 ± 0.5	3.8 ± 0.4	49.0 ± 9.5	44.0 ± 14.3
	12	101.0 ± 7.3	18.0 ± 2.6	0.70 ± 0.15	7.10 ± 0.77	3.8 ± 0.5	3.3 ± 0.3	68.0 ± 6.4	35.0 ± 9.8
Placebo	0	110.0 ± 12.7	18.0 ± 2.7	0.60 ± 0.17	8.40 ± 0.82	3.6 ± 0.5	4.8 ± 0.3	57.0 ± 10.1	42.0 ± 6.6
	4	104.0 ± 9.4	17.0 ± 1.9	0.70 ± 0.08	7.30 ± 0.81	3.7 ± 0.5	3.6 ± 0.3	50.0 ± 12.6	36.0 ± 8.9
	8	99.0 ± 6.1	18.0 ± 2.0	0.60 ± 0.11	7.90 ± 0.74	3.8 ± 0.3	4.1 ± 0.4	41.0 ± 12.8	50.0 ± 13.0
	12	106 ± 12.9	19.0 ± 4.1	0.60 ± 0.10	6.80 ± 0.76	3.7 ± 0.3	3.1 ± 0.4	64.0 ± 10.7	33.0 ± 11.3
Gluc	0	107.0 ± 9.6	18.0 ± 2.9	0.70 ± 0.12	6.60 ± 0.91	3.3± 0.5	3.3 ± 0.4	65.0 ± 5.0	37.0 ± 14.5
	4	99.0 ± 13.5	21.0 ± 4.3	0.60 ± 0.14	6.80 ± 0.81	3.2± 0.5	3.6 ± 0.3	49.0 ± 6.8	47.0 ± 6.2
	8	100.0 ± 9.2	20.0 ± 3.1	0.50 ± 0.23	8.30 ± 0.73	3.6± 0.41	4.7 ± 0.3	52.0 ± 10.0	38.0 ± 6.5
	12	103.0 ± 12.2	19.0 ± 2.7	0.60 ± 0.14	7.60 ± 0.83	3.3± 0.3	4.3 ± 0.5	63.0 ± 7.2	37.0 ± 13.0
LL	0	102.0 ± 11.7	18.0 ± 3.3	0.70 ± 0.18	7.70 ± 0.82	3.8± 0.4	3.9 ± 0.4	42.0 ± 9.2	37.0 ± 12.2
	4	95.0 ± 8.6	20.0 ± 3.1	0.60 ± 0.16	7.80 ± 0.81	4.0 ± 0.3	3.8 ± 0.5	52.0 ± 10.1	47.0 ± 8.9
	8	94.0 ± 12.4	19.0 ± 3.5	0.70 ± 0.19	8.40 ± 0.94	3.9± 0.5	4.5 ± 0.5	57.0 ± 5.9	33.0 ± 12.0
	12	109.0 ± 10.8	18.0 ± 3.6	0.60 ± 0.15	7.40 ± 0.84	3.9± 0.4	3.5 ± 0.5	59.0 ± 13.2	38.0 ± 8.9

Gluc=Glucosamine, LL=*Lilium lancifolium*, GLU=Glucose, BUN=Blood urea nitrogen, CREA=Creatinine, TP=Total protein, ALB=Albumin, GLOB=Globulin, ALT=Alanine aminotransferase, ALP=Alkaline phosphatase

### Clinical lameness score

**Figure-2:**
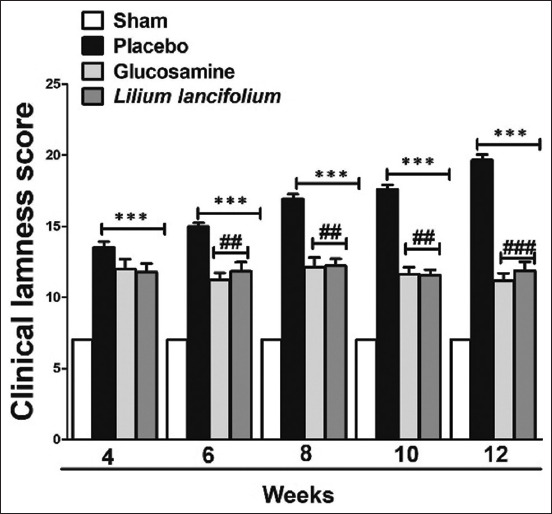
Effect of *Lilium lancifolium* on clinical lameness scores of all the experimental groups. The values represent the mean ± SEM of the total score (n = 4). ***p < 0.001 versus sham group, ^##^p < 0.01 and ^###^p < 0.001 versus placebo group. SEM=Standard error of the mean.

As indicated in Figure-2, clinical lameness scores significantly increased in placebo, glucosamine, and *L. lancifolium* groups compared with those in the sham group from weeks 4 to 12. However, the lameness scores eventually decreased in the glucosamine and *L. lancifolium* groups at weeks 6, 8, 10, and 12 after the first treatment compared with the placebo group. This condition lasted until week 12, when the clinical lameness scores of the *L. lancifolium* group decreased by approximately 8 ± 0.8 compared with those of the placebo group. No significant difference was observed between the glucosamine and *L. lancifolium* groups from week 4 until the end of the experiment.

### Radiographic assessment

**Figure-3:**
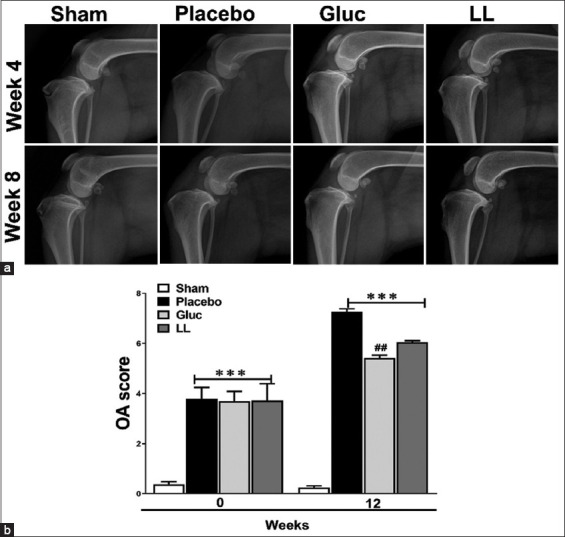
Radiographic evaluation of all dogs before and after treatment. Radiography (a) and Comparison of radiographic osteoarthritis severity scores (b) between different groups. The values represent the mean ± SEM of the total score (n = 4). ***p < 0.001 versus sham group, ^##^p < 0.01 versus placebo group. LL=*Lilium lancifolium*, OA=Osteoarthritis, SEM=Standard error of the mean.

X-rays are commonly used to assess the OA’s severity [30]. To evaluate the therapeutic effect of *L. lancifolium* in decreasing the severity of OA, radiographic analysis was conducted after OA induction and at week 12 of the experiment. Severity scores were significantly higher in the placebo, glucosamine, and *L. lancifoliu*m treatment groups than the sham group, as appears in the radiographic (Figure-3). However, at each time point, glucosamine exhibited significantly lower OA scores than the placebo group. Although *L. lancifolium* showed a reduction in the OA scores, the differences in severity were not statistically significant compared with those of the placebo group (Figure-3).

### Anti-inflammatory cytokines

**Figure-4:**
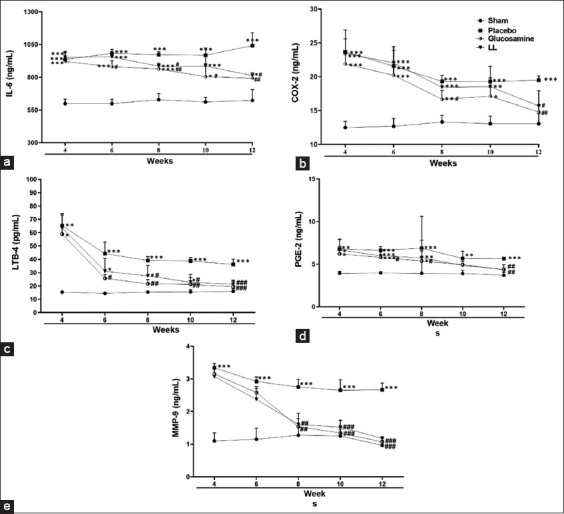
Effects of *Lilium lancifolium* on inflammatory cytokines and mediators in Osteoarthritis-induced dogs. The values represent the mean ± SEM of the total score (n = 4). *p < 0.05, **p < 0.01, and ***p < 0.001 versus sham group, ^#^p < 0.05, ^##^p < 0.01, and ^###^p < 0.001 versus placebo group. IL-6=Interleukein-6, cox-2=Cyclooxygenase, LTB-4=Leuktorine-4, PGE-2=Prostaglandin E-2, MMP-9=Metalloproteinases-9, SEM=Standard error of the mean.

Inflammatory cytokines play a destructive role in the cartilage and are eventually involved in the pathophysiological progression of OA [7]. Therefore, the effects of *L. lancifolium* on changes in the serum levels of pro-inflammatory cytokines and inflammatory mediators, namely, IL-6, COX-2, LTB-4, PGE-2, and MMP-9, were examined. The placebo group showed significantly increased levels of cytokines and mediators compared with those in the sham group (p < 0.05), as shown in Figure-4.

An increase in the level of IL-6 induced a significant acute immune response in the placebo group. This was prevented in the glucosamine-treated group at 6, 8, 10, and 12 weeks (p < 0.05–0.01). Furthermore, the acute immune response was significantly decreased in the *L. lancifolium*-treated group compared with the placebo group at weeks 8 and 12 (p < 0.01). Besides, COX-2 levels decreased significantly at weeks 8 and 12 in the glucosamine group and week 12 in the *L. lancifolium* group compared with those in the sham group, as shown in Figure-4b.

The levels of LTB-4 exhibited a time-dependent decreasing pattern. Glucosamine treatment significantly minimized the levels of the LTB-4 (p < 0.05–0.001) compared with those of the placebo group; besides, the *L. lancifolium*-treated group indicated a significant reduction in the level of LTB-4 compared with that of the placebo group at weeks 8, 10, and 12 (p < 0.05–0.001), as indicated in Figure-4c. Furthermore, PGE-2 release in the serum was inhibited significantly in the glucosamine- (weeks 6–12) and *L. lancifolium*-treated groups (week 12) compared with that in the placebo group (p < 0.05–0.01), as shown in Figure-4d. Moreover, MMP-9 is well known for its role in destroying joints [31]. Figure-4e indicates that glucosamine and *L. lancifolium* therapies significantly decreased the levels of MMP-9 at weeks 8, 10, and 12 versus the placebo group (p < 0.05–0.001). The results in Figure-4 show that *L. lancifolium* has an immunomodulatory effect in the OA dog model.

### Histologic changes in articular cartilage

**Figure-5:**
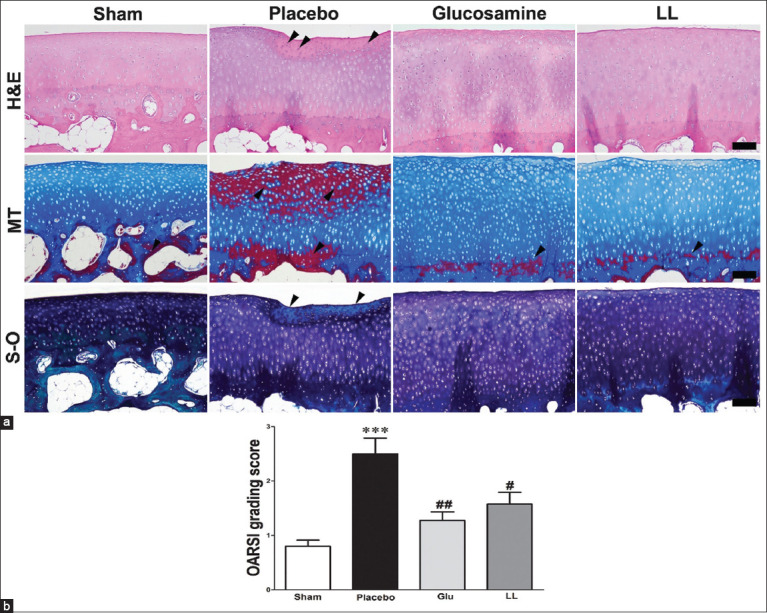
Microscopic assessments of articular cartilage. Microscopic lesions of the articular cartilage (scale bar = 100 μm) in all experimental groups (a) OARSI grading score of articular cartilage damage (b). ***p < 0.001 versus sham group, ^#^p < 0.05 and ^##^p < 0.01 versus placebo group. LL=*Lilium lancifolium*, OARSI=Osteoarthritis Research Society International, H&E=Hematoxylin and eosin, MT=Masson’s trichrome, S-O=Safranin-O.

Histopathological assessments of articular cartilage were conducted after staining with H&E, MT, and S-O fast green. The placebo group showed the destruction of the articular cartilage surface and inflammatory cell infiltration compared with the sham group. By contrast, inflammatory cell infiltration was decreased, and articular cartilage destruction tended to be suppressed in the glucosamine and *L. lancifolium* groups in the H&E slides (Figure-5a). The difference in the collagen content of articular cartilage between groups after MT staining was determined in the placebo group. A tidemark was found with the collagenization of the articular cartilage (superficial and mid-zone). However, in the glucosamine and *L. lancifolium* groups, collagenization was verified only in the tidemark, as shown in the MT slides (Figure-5). The S-O stain results (Figure-5) showed that the placebo group had more tissue damage than the other groups. OARSI score was measured in all groups of articular cartilage. The placebo group indicated a significantly increased OARSI score compared to the sham. By contrast, OARSI scores were substantially decreased in the *L. lancifolium* (p < 0.05) and glucosamine (p < 0.01) groups compared with that of the placebo group, as indicated in Figure-5b.

## Discussion

This study evaluated the efficacy of *L. lancifolium* in alleviating inflammatory cytokines and histological changes in an OA canine model induced by CrCL and LCL resection. Furthermore, functional assessment methods involved clinical lameness score (including the severity of pain), radiographic evaluation, and ELISA during the 12-week study period. The results show that histological damage and the release of inflammatory cytokines (IL-6, COX-2, LTB-4, PGE-2, and MMP-9) caused by OA induction were significantly decreased in the glucosamine and *L. lancifolium* extract groups. NSAIDs are currently commonly used to treat OA. However, several studies have found that long-term use of NSAIDs causes various health problems, including nephrotoxicity [32, 33] and gastrointestinal disorders [26, 34]. These preventive and alternative solutions are nutritional, particularly herbal medicines and dietary supplements. Recently, the role of many dietary supplements in alleviating OA in dogs has been examined [35]. Hence, *L. lancifolium* extract was chosen in our study for its anti-inflammatory and antioxidant effects [27].

It has been reported that patients with OA experience pain, which causes lameness and functional impairment [36]. Our result in Figure-2 is consistent with this finding, shows that the lameness score is high in the untreated group but low in the *L. lancifolium*-treated group. The reduction in clinical lameness score following *L. lancifolium* treatment was observed 2 weeks after *L. lancifolium* administration. At week 12, the end of the experiment, the clinical lameness score of the *L. lancifolium* group was 12 ± 1.4, which showed an improvement of 8 ± 0.8 compared with the placebo group. These effects are attributed to the anti-inflammatory effects of the *L. lancifolium* extract and its richness in antioxidants [37].

Although radiographic analysis showed a decrease in the OA score but was insignificant when compared with the placebo group (Figure-3). However, the improvement in histological damage indicated in Figure-4 could be the start of the treatment progression. Clinical manifestations of OA occur because of damage to the collagen matrix in cartilage, which disrupts the overall bone structure and causes joint pain [38, 39]. This conclusion is consistent with our findings in Figure-5, where MT and S-O staining validated the proteoglycan damage and fibrosis in the placebo group. Furthermore, histological examination showed that the glucosamine and *L. lancifolium* groups preserved more articular cartilage than the placebo group. Because OA is a chronic disease with longer therapeutic progression, particularly in older dogs, this positive effect will be reflected in clinical improvement over time [40]. Therefore, it is anticipated that improvements in lameness and radiographic OA scores will take 6 months or longer.

Interestingly, our results (Figure-4) indicated that the inflammatory cytokines eventually improved. When compared with the placebo group, glucosamine, and *L. lancifolium* extract treatments significantly decreased the levels of IL-6, COX-2, LTB-4, PGE-2, and MMP-9 over the study period. Inflammatory cytokines are well known to play a role in OA’s progression. Wojdasiewicz *et al*. [7] previously classified IL-6 as a strong immune system stimulant manufactured in response to other inflammatory mediators that is highly expressed in synovial fluid and serum and is positively correlated with the intensity of lesions exhibited in X-ray imaging. Our findings are also consistent with a recent study of OA in humans, which showed that osteoarthritic osteoblasts and articular chondrocytes possess high levels of COX-2, LTB-4, PGE-2, and MMP-9 [41, 42]. COX-2 converts arachidonic acid to PGE2, which causes pain by signaling to peripheral receptors. Arachidonate 5-lipoxygenase produces LTB4, which promotes the synthesis and release of pro-inflammatory cytokines from the synovial membranes [32]. Hence, the inhibitory activities of *L. lancifolium* extract on these cytokines enhanced the surgically induced inflammatory reactions by the OA model.

MMP-9 levels were lower at weeks 6 and 8 in response to *L. lancifolium* treatment compared with control animals, according to our findings (Figure-4). MMP-9 is a member of the MMP family essential in OA pathogenesis [43]. MMPs degrade collagen, proteoglycans, and other extracellular matrix macromolecules in cartilage. Consequently, the insult to tissue is OA, which starts with MMP-9 production [44]. Because of these findings, it is assumed that *L. lancifolium* improves OA effects by inhibiting the release of many cytokines, most notably MMP-9.

## Limitations

The study is too small, and the trial may not be long enough to assess the effects of *L. lancifolium* extract on clinical lameness. It is also restricted to old male dogs; however, this is justified given the nature of the disease, which primarily affects the elderly. Computed tomography scans were not used to examine the specifics of treatment progression. Furthermore, the molecular mechanism underlying *L. lancifolium*’s effects on OA-associated inflammatory reactions was not assessed. These issues appear to provide a useful framework for additional research.

## Conclusion

In this study, changes in inflammatory parameters and clinical condition in the joint were confirmed after CrCL/LCL resection-induced OA in canine models. *L. lancifolium* attenuated inflammation and improved histological damage. Both glucosamine and *L. lancifolium* indicated anti-inflammatory effects in dogs with OA. This effect was very similar to that of glucosamine, which is used for OA treatment. Supplementation with *L. lancifolium* may represent management and potential therapeutic options in dogs with OA. However, long-term and large-scale clinical trials are required to establish the potential long-term benefits of *L. lancifolium* used in this study.

## Authors’ Contributions

HO and JC: Conceived and planned the study. JC, YP, and JiC: Performed the study. JC and GOA: Analyzed the data. JC, GOA, HP, and HO: Drafted and revised the manuscript. EJ, HP, and HO: Collected the data and followed up the animals. All authors have read and approved the manuscript.
